# Human-elephant conflict in western Thailand: Socio-economic drivers and potential mitigation strategies

**DOI:** 10.1371/journal.pone.0194736

**Published:** 2018-06-01

**Authors:** Antoinette van de Water, Kevin Matteson

**Affiliations:** 1 Department of Biology, Miami University, Oxford, Ohio, United States of America; 2 Bring the Elephant Home Foundation, Chiang Mai, Thailand; Sichuan University, CHINA

## Abstract

Understanding human-wildlife conflict is an important first step in the conservation of highly endangered species that can have adverse effects on human communities, such as elephants. To gain insights into variables that shape attitudes toward elephant conservation in Asia, we surveyed 410 households and 46 plantation owners in seven villages around the Salakpra Wildlife Sanctuary in western Thailand, an area of high human-elephant conflict. We sought to evaluate how past experiences with elephants (positive or negative), as well as socio-economic variables (age, income level, gender, and employment type) affect attitudes toward elephant conservation and coexistence in this area. In addition, we quantified deterrence methods currently used and identify potential mitigation strategies supported by community members. In general, less supportive attitudes toward elephant conservation and coexistence were held by individuals older than 35 years of age, those who had previously had experienced negative interactions with elephants, those with lower incomes, and those working in the agricultural sector. Conversely, those who had received benefits from living near elephants (e.g., supplemental income or feelings of pride from hosting volunteers or participating in conservation work) had more supportive views of elephant coexistence. Plantation owners reported using a variety of deterrence methods with varying success, with firecrackers being the most commonly utilized method. Community members identified several potentially beneficial mitigation strategies including forest restorations and patrol teams, adding water sources to wild elephant habitat, and education of local school and community groups. Overall, our results highlight the value of community members receiving benefits from living near elephants and suggest that special incentives may be needed for demographic groups disproportionately affected by elephants (e.g. those at lower income levels, those working in agriculture). A combination of these and other approaches will be required if human-elephant coexistence in western Thailand is to be realized.

## Introduction

Alleviation of human-wildlife conflicts is one of the great challenges for conservation of wildlife worldwide [1,2]. Several studies have demonstrated that people are more willing to tolerate and coexist with wild species when they receive benefits from generating income through tourism, a pleasure to see wildlife, feeling more secure/safe, and ecological services provided by wildlife [3,4]. However, conflicts with individual animals can lead to persistent negative views of ‘problem’ species [5], reducing the likelihood of future conservation efforts [6]. In addition to direct experiences (positive or negative) with wildlife, socio-economic variables such as income level, gender, and employment type can all influence community perception of wildlife [3,4]. A detailed understanding of community perceptions of human-wildlife conflicts is necessary to facilitate coexistence of humans and wildlife in many areas of the world.

Reduction of human-wildlife conflict is especially challenging for large, potentially dangerous and damaging species such as elephants. In 2008, an estimated 38,500–52,500 wild Asian elephants (*Elephas maximus*) were remaining globally, primarily in India, Myanmar, Thailand, Sri Lanka, Malaysia and Indonesia [7,8]. In this region, habitat destruction and fragmentation often result in increasing conflicts between people and elephants [8,9]. Although Asian elephants are listed by the IUCN as endangered, [10] human-elephant conflict (HEC) continues to result in hundreds of elephant and human deaths per year [11], and HEC is considered a major threat to the future of Asian elephants [8,12]. Identifying the factors that contribute to HEC is a critical step towards effective conservation of Asian elephants.

A variety of socio-economic factors have been shown to influence attitudes towards HEC and elephant conservation. In Kenya [4] and Uganda [13], women were slightly less tolerant of elephants or elephant conservation initiatives than men, potentially due to their greater involvement in agricultural activities to sustain their families, an employment type considered to be threatened by elephants [13]. Supporting this idea, people’s employment type has been shown to influence perceptions of elephants in Kenya, with people working in agriculture exhibiting less tolerance of elephants than people working in other areas [4]. Alternately, those receiving benefits from the presence of elephants (e.g. via ecotourism, employment, enjoyment to see elephants) have a higher inclination to support conservation [3,4,14]. In India, about 50% of the survey participants stated that women were equally affected by human-elephant conflicts, even though women were carrying a disproportionate burden of these effects [15]. Gender, age, and income have also shown to play a role in shaping attitudes toward conservation of species that contribute to human-wolf conflict in India, with age showing a negative correlation with attitudes, males having more positive attitudes and the number of income sources having a positive association with attitudes toward wolves [16].

Although roughly half of the geographic range of elephant habitat in Thailand is considered suitable for long-term elephant conservation, much of this area is threatened by agriculture, roads and other development [17] resulting in fragmentation and increased HEC [18]. The Tenasserim range, along the border between Thailand and Myanmar, is a priority area for the conservation of the Asian elephant and one of the last places in Asia with enough continuous habitat to support viable elephant populations [17]. Salakpra Wildlife Sanctuary on the Tenasserim range is home to an estimated 181 Asian elephants [19]. Increased agricultural activity, growing human populations, the construction of roads and of the Tha Tung Na and Srinagarind dams, have all contributed to habitat fragmentation in and around this protected area [19]. These factors have resulted in increasing HEC [20], which now causes a variety of problems for the human communities in the area while also threatening the long-term survival of the elephant population [18].

**Fig 1:**
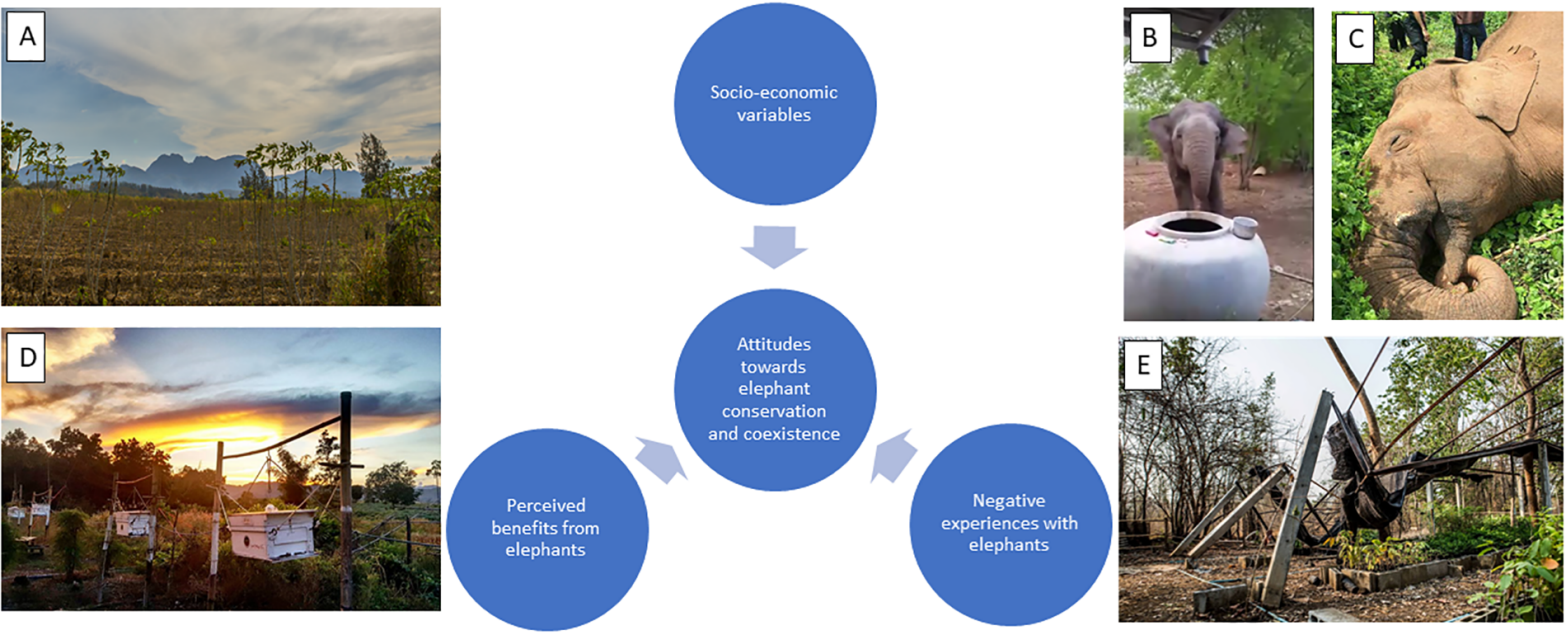
Examples of human-elephant conflict in the Chong Sadao district, Thailand as well as general hypotheses on factors affecting perceptions of elephant conservation and coexistence. Panels show A) a cassava plantation that is frequently raided by wild elephants, B) a wild elephant searching for water, C) a wild male elephant electrocuted by electric fencing on a corn plantation, D) a sustainable elephant deterrence method that provides benefits, and E) a community tree nursery severely damaged by elephants.

The overall purpose of this paper is to improve understanding of the variables that influence attitudes toward Asian elephant conservation, with a goal of mitigating conflicts and promoting human-elephant coexistence. We explored perceptions of HEC for 410 households and 46 plantations distributed in seven villages around the Salakpra Wildlife Sanctuary in western Thailand, examining trends in crop and property damage, as well as feelings about existing and potential methods for reducing HEC. We assessed several hypotheses regarding the relationship between socio-economic variables and perceptions of elephants in the area. Specifically, we evaluated how socio-economic variables (gender, age, employment sector, income), negative experiences with elephants (e.g., crop raiding, property damage), or perceived benefits from elephants (e.g., income from ecotourism, involvement in community conservation) affect residents’ stated support for elephant conservation and willingness to tolerate elephants in the area (Fig 1). We predicted that socio-economic variables, as well as past experiences with elephants, would play a large role in structuring community feelings toward elephant conservation. The results in this paper represent the first detailed study of perceptions of HEC in villages around the Salakpra Wildlife Sanctuary in Thailand and contribute to a broader understanding of HEC in Asia.

## Methods

### Study area

**Fig 2:**
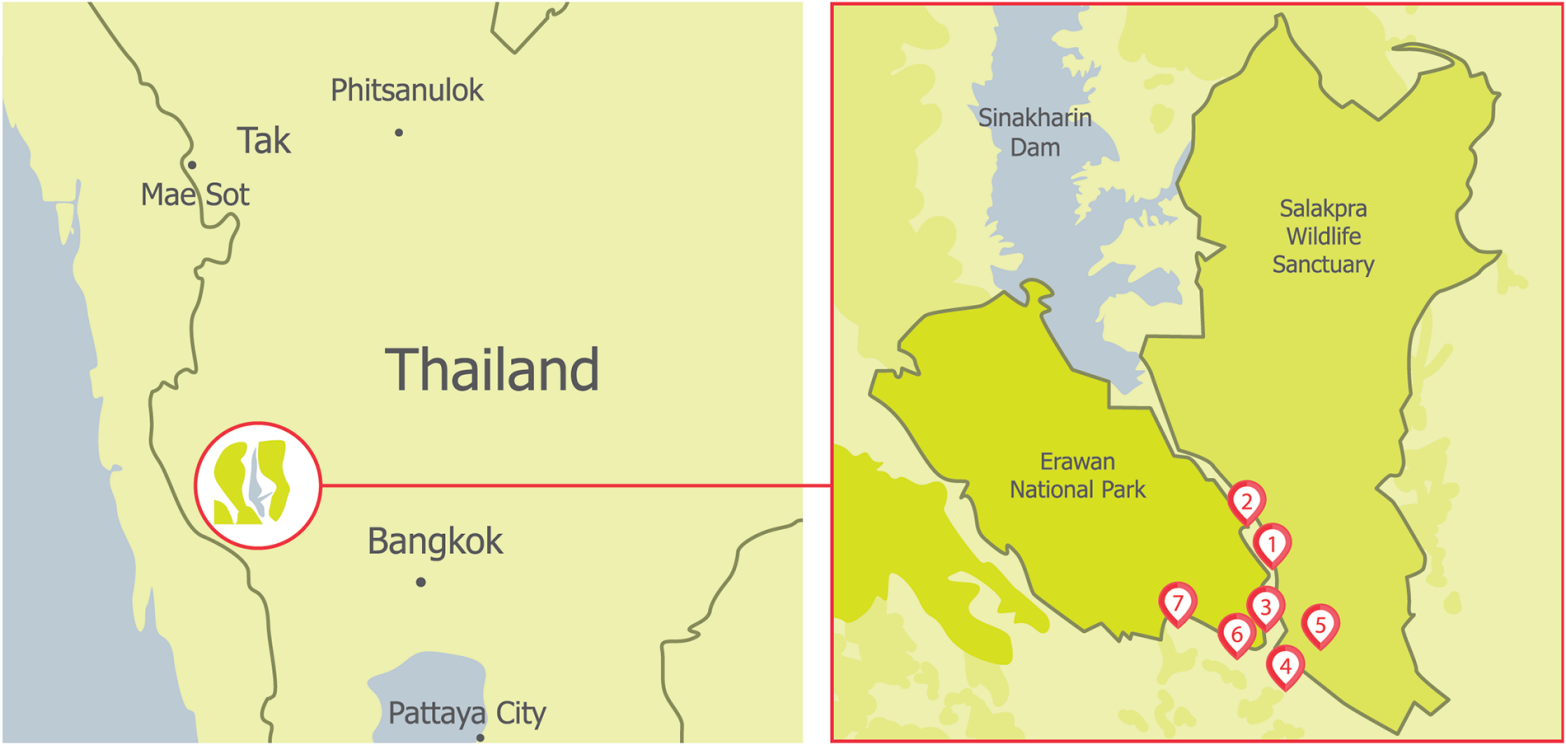
Relative locations of Salakpra Wildlife sanctuary and the seven participating villages in the area of high human-elephant conflict. (1) Chong Sadao), (2) Ban Mo Thao), (3) Ban Pong Pat, (4) Ban Chong Krathing, (5) Ban Kang Pla Kod), (6) Ban Nong Pra Chum and (7) Ban Tub Sila. Adapted from Google Maps by A. van de Water.

The Chong Sadao district in western Thailand consists of seven villages with a combined population of 3,649 people. The district is located on the banks of the Kwai Yai River and borders both Salakpra Wildlife Sanctuary and Erawan National Park (Fig 2). The landscape outside the protected areas is dominated by cassava and sugarcane plantations and is adjacent to the Khao Chong Krathing and Khao Tha Manao mountains. The district is an area of high human-elephant conflict [20], especially during the dry season (February to June) when elephants search for water sources located outside the protected areas (W. Sujirat, personal communication, 2015).

### Data collection

The study consisted of two questionnaires, a primary questionnaire aimed at households in the region (‘household questionnaire’ hereafter), and a follow-up questionnaire that specifically targeted plantation owners (‘plantation owner questionnaire’, hereafter) because of their vulnerability to elephant-induced damage. The household questionnaire included 14 questions (S1 Appendix) focused on attitudes and perceptions of villagers towards elephants. The questionnaire was translated into Thai and was administered in October 2015 to 410 households. We first approached village leaders to explain the objectives of the research, who then helped facilitate distribution of the questionnaires to the randomly selected households. All community members that were present at the moment of the questionnaire were given an opportunity to fill out the questionnaire. We estimate that ~38% of total households in Chong Sadao district participated in the household questionnaire, with only one participant per household. Participation was completely voluntary and no incentives (financial or other) were provided for filling out the questionnaire. In March 2016, the 12-question plantation owner questionnaire (S2 Appendix) was translated into Thai and was administered to gain a deeper insight into the attitudes of 46 plantation owners. Plantations were defined as land used to grow cash crops with a minimum size of one rai (1600 m^2^). The average size of the plantations was 24.4 rai (39,040 m^2^). It is possible that some of the participants in the plantation owner questionnaire also participated in the household questionnaire. Both questionnaires were reviewed and approved by the Miami University Internal Review Board with project reference number 01805e. Informed consent, written in Thai, was obtained before participants enrolled in the study. Both questionnaires were divided into two sections to gain insight into (i) socio-economic variables and past experiences with elephants (positive or negative) and (ii) attitude toward and perception of potential solutions. The household questionnaire included demographic questions and focused on the impact of HEC at the household level, including people’s overall perceptions of elephants in the community. The plantation owner questionnaire sought to examine what types of crops are usually raided, the level of crop raiding, currently used elephant deterrence methods and their perceived efficacy.

### Data analysis

We used logistic regression (SPSS 24, SPSS, Inc., Chicago, USA) to evaluate how socio-economic variables (gender, employment type, age, and income) and past experiences with elephants (negative or positive) affect respondents’ ‘attitude toward elephant conservation’ and ‘attitude toward human-elephant coexistence’. Employment was grouped into those working in agriculture versus all other non-agricultural work areas. Our survey asked for participant age according to six possible age range categories (18–24 years old, 25–34 years old, 35–44, 45–54, 55–64, and 65 and older). To increase statistical power and clarity of recommendations, age was grouped into two categories for statistical analyses (<35 years old versus 35 and older). Similarly, income was analyzed as less than 10,000 THB (301 USD) and greater than or equal to 10,000 THB per month.

‘Attitude toward elephant conservation’ was based on the question “Do you feel it is important to invest in elephant conservation?” for which respondents could answer “yes” or “no”. The respondents also had the option to specify the reasoning behind their answer (see S1 Appendix) but, for the purposes of this paper, we focused just on the frequency of “yes” and “no” responses. For ‘attitude toward human-elephant coexistence’ we analyzed responses to the question “Which statement describes your attitude toward elephants most accurately?” which included three possible response categories: 1) “I tolerate elephants in my environment”, 2) “I would tolerate elephants in my environment if the elephants would stop destroying my plantations” and 3) “I would prefer the elephants to be eradicated”. For simplicity, we condensed these responses to 1) “tolerate”, 2) “conditional tolerate”, and 3) “eradicate”. To evaluate the dichotomous dependent variable ‘attitude toward elephant conservation’, we used binary logistic regression, with a Hosmer and Lemeshow Test used to evaluate overall model fit. For ‘attitude toward human-elephant coexistence’, with three categories, we used multinomial regression with a likelihood ratio test used to evaluate the model fit. Variables were removed if not statistically significant at an alpha of 0.05. Interactions were not included to keep the set of variables to a reasonable size and due to a lack of clear *a priori* hypotheses regarding the influence of interactions.

We used the plantation owner survey to evaluate the frequency and types of HEC occurring on plantations. We generally compared plantation owner perceptions of elephants to verify the differences between employment sectors found in the household questionnaire. Finally, we summarized the current deterrence methods utilized by plantation owners and the perceived efficacy of these methods. The question about the deterrence methods utilized by plantation owners had a category for ‘other methods’ to account for used methods outside of the categories we had predefined as deterrent methods. From field experience, we have not heard that people were using ginger or chili in the area of this study, so these methods were not included as a predefined category in the plantation questionnaire.

## Results

### Household questionnaire

Of the 410 participants in the household survey, 51.5% of respondents were female, 29.5% of participants were under 35 years of age and 51.5% were between 35 and 54 years old. 25.5% of households were located within 750 m of the protected area boundary. Three respondents answered they lived inside the protected area. In addition, although one village appears to be located within the boundaries of the protected area (Fig 2), it is important to note that the boundaries between the protected area and the community forest in this area are not entirely clear. The main source of reported income was general labor (44.1%, including manufacturing, construction, and retail trade) followed by those working in agriculture (22.5%), and ‘other’ types of income (20.4%, including accommodation and food services and support from adult children and others). 9.1% of respondents worked as officials (including public administration, education, and public health services) and 3.8% stated that their main income came from forest products. Income was relatively low in the area of our study: 59.9% of the participants declared incomes of less than 10,000 THB (301 USD) per month and 37.4% declared incomes of between 10,000 THB (301 USD) and 20,000 THB (602 USD) per month. The national average monthly wage for Thailand in 2016 was 413 USD (National Statistical Office of Thailand).

Just over half (52.7%) of the surveyed households reported experiencing a negative impact from HEC over the last two years (October 2013–October 2015). Of those who reported a negative experience, 104 respondents (48.4%) provided additional details regarding the type of negative impact. The most commonly identified type of negative effect of elephants was property damage (49.0%) (e.g., see Fig 1, Panel E). Twenty-four respondents (23.1%) mentioned that they saw elephants passing the house, crossing the road or drinking water, although it was not clearly stated how, exactly, this was a negative interaction. Wild elephants have killed people in this area so it is reasonable for a wild elephant sighting to elicit fear and to potentially be a negative experience. Ten respondents (9.6%) reported experiencing crop raiding by elephants. Nine respondents (8.7%) reported fear from seeing elephants, and six respondents (5.8%) mentioned the inconvenience of traveling when elephants are near the house. Two respondents (1.9%) answered that they had experienced human injuries caused by elephants, and an additional two individuals did not specify the negative impact they experienced from elephants nearby.

Just over a third of respondents (35.9%) reported a perceived benefit of living near wild elephants. The exact nature of these benefits varied, with 46.6% indicating community development, 24.4% indicating a feeling of pride from hosting conservation volunteers (e.g. volunteers to plant trees), 17.6% indicating a feeling of satisfaction/pride to do conservation work, 8.3% reporting financial benefits through conservation jobs, and 3.1% reporting financial benefits due to ecotourism jobs.

#### Influence of socio-economic variables on attitudes toward elephant conservation and coexistence

Attitudes toward elephant conservation and coexistence varied by socio-economic variables (S3 Appendix). We found no significant difference in overall attitude toward elephant conservation by gender, income or employment type (those working in agriculture versus those not working in agriculture). We did, however, find a significant difference in the overall attitudes toward elephant conservation by age (Table 1), with residents older than 35 years of age being less likely to view elephant conservation as important than their younger counterparts (coefficient = -0.783 [SE 0.372], p = 0.035). In addition, those who had experienced a negative impact of elephants were significantly less likely to view elephant conservation as important (coefficient = -1.184 [SE 0.328], p < 0.001; Table 1). The binary logistics model including age and negative experiences with elephants fit the data well (Hosmer and Lemeshow Test = 0.568, df = 2, p = 0.753).

**Table 1:** Logistic regression results showing significant variables influencing residents’ attitude toward elephant conservation.

Variable	B (S.E.)	Wald	Exp(B) (95% C.I.)	P-value
Age	-0.783 (0.372)	4.429	0.457 (0.220–0.035)	0.035
Experienced a negative impact from elephants	-1.184 (0.328)	13.067	0.306 (0.161–0.582)	<0.001
Constant	-1.088 (0.182)	35.619	0.337	<0.001

When evaluating feelings towards coexistence with elephants (preference for tolerance, conditional tolerance, or local eradication), we found no difference by gender or age. There was, however, a difference in attitude toward human-elephant coexistence by employment type and income, with those working in agriculture (coefficient = -1.131 [SE 0.376], p = 0.003; Table 2) or making less than 10,000 THB/year (coefficient = -0.724 [SE 0.271], p = 0.008; Table 2) being less likely to unconditionally tolerate elephants. In addition, those who had not experienced a negative effect of living near elephants were more likely to support both eradication (coefficient = 0.695 [SE 0.260], p = 0.007) and unconditional tolerance of elephants (coefficient = 0.959 [SE 0.282], p = 0.001), an unexpected result. Finally, those who had not received benefits from living near elephants were more likely to support eradication of elephants from the area (coefficient = 1.005 [SE 0.296], p = 0.001; Table 2). The multinomial logistics model including the above variables fit the data well (Likelihood Ratio Test = 72.204, df = 8, p < 0.001).

**Table 2:** Multinomial logistic regression results showing significant variables influencing residents’ attitude toward elephant coexistence.

Variable	B (S.E.)	Wald	Exp(B) (95% C.I.)	P-value
Eradicate				
Employment (Agriculture)	0.369 (0.269)	1.883	1.446 (0.854–2.449)	0.170
Income (<10,000 THB)	-0.407 (0.261)	2.436	0.665 (0.399–1.110)	0.119
Negative impact of HEC (No)	0.695 (0.260)	7.181	2.005 (1.205–3.334)	0.007
Perceived benefits (No)	1.005 (0.296)	11.498	2.731 (1.528–4.881)	0.001
Intercept	-1.003 (0.304)	10.903		0.001
Tolerate				
Employment (Agriculture)	-1.131 (0.376)	9.063	0.323 (0.154–0.674)	0.003
Income (<10,000 THB)	-0.724 (0.271)	7.132	0.485 (0.285–0.825)	0.008
Negative impact of HEC (No)	0.959 (0.282)	11.539	2.609 (1.500–4.536)	0.001
Perceived benefits (No)	-0.421 (0.281)	2.242	0.656 (0.378–1.139)	0.134
Intercept	0.123 (0.256)	0.232		0.630

“Conditional tolerance” was set as the reference category and departures towards “eradicate” and “tolerate” are shown below. Gray shading indicates significance at p < 0.05.

#### Potential HEC mitigation methods preferred by household owners

The potential HEC mitigation methods deemed essential by the respondents of the household questionnaire were ‘Forest restoration efforts inside the wildlife sanctuary’ and ‘Patrol team to chase elephants back into the protected area’. Respondents also frequently reported ‘Forest restoration efforts at the buffer zone” as a high priority. Support for most mitigation actions was high. For example, 98% of respondents felt that “Initiatives to realize benefits for local communities” and “Education of local groups and schools” were medium, high, or essential priorities (Table 3).

**Table 3:** Priorities for mitigating human-elephant conflict as ranked by 410 household residents in the Chong Sadao district of western Thailand in October 2015.

Potential community-wide human-elephant conflict mitigation strategies ranked by importance	Essential	High priority	Medium priority	Low priority	Not a priority
Forest restoration efforts inside the wildlife sanctuary	36.1%	48.0%	9.0%	1.0%	5.9%
Patrol team to chase elephants back into the protected area	31.5%	38.5%	25.4%	4.1%	0.5%
Fencing the wildlife sanctuary so that the elephants cannot get out	26.6%	46.8%	22.9%	2.7%	0.0%
Forest restoration efforts at the buffer zone	24.9%	52.9%	17.1%	2.7%	1.5%
Adding water sources to wild elephant habitat (check dams, reservoirs)	22.4%	44.6%	28.8%	1.7%	2.4%
Initiatives to realize benefits for local communities (ecotourism, conservation jobs)	20.2%	54.4%	23.4%	0.7%	1.5%
Education of local groups and schools	17.3%	52.9%	28.0%	1.2%	0.5%

### Plantation owner questionnaire

The plantation owner questionnaire was completed by 46 respondents. We did not collect detailed demographic data on plantation owners (e.g., age, income), but the majority of plantations were family-owned and ownership was quite evenly divided between men and women. All plantations were within 4 km of the protected areas. The largest plantations were primarily used to grow cassava and sugarcane, while other cash crops included banana, jackfruit, mango, tamarind, lime and kaffir lime.

#### Causes and impacts of HEC on plantations

Just over half of plantation owners (54.3%) stated that their plantations are raided by elephants daily. Nearly all (95.7%) experienced crop raiding at least once a month. All sugarcane plantation owners (8 respondents) reported that their plantation had been raided by elephants at least once a week. The major crops mentioned by the participants as raided by elephants were cassava (41.5%, *n* = 200), banana (17.0%), jackfruit (10.0%), mango (8.5%) and tamarind (3.5%). Plantation owners estimated annual losses to be less than 10,000 THB (301 USD) per year in 52.2% of these cases, while 28.3% estimated it to be between 10,000 and 20,000 THB (301–602 USD). Although we did not collect data on financial loss due to property damage and human injury, the financial burden of these incidences is substantial and likely higher than crop damage costs in most cases (e.g., in 2018, replacement of a water tank is estimated to cost at least 9,000 THB and a new water pipe system can cost more than 6,000 THB). Furthermore, human injuries can result in catastrophic financial losses if an individual becomes unable to work and incurs medical expenses. Property damage by elephants other than crop raiding was mentioned by 71.7% of plantation owners and human injury was reported by 2.2% of plantation owners. 26.1% of the plantation owners reported that elephants did not cause any harm other than crop raiding. The most commonly reported property damage was breaking of water pipes and water tanks (63.0%).

69.6% of the participants in the plantation owner questionnaire wanted elephants eradicated, a much higher proportion than in the household survey (34.3%).

#### Deterrence methods currently used by plantation owners

The most common method reported by plantation owners to deter elephants was firecrackers (used by 87.0% of respondents), and firecrackers were also considered the most effective method (Table 4). Less than half of plantation owners used electric fencing, which was largely considered semi-effective (63.6%) or not effective (31.8%). Elephant watchtowers and non-electric fences were less-utilized and considered less effective. Other deterrence methods that were anecdotally mentioned include voice/shouting, calling park officers, and use of dogs, fire, flashlights or cars to scare or chase elephants.

**Table 4:** Elephant deterrence methods and their perceived efficacy as reported by 46 plantation owners on the western boundaries of Thailand’s Salakpra Wildlife Sanctuary in October 2016.

Elephant deterrence methods used by plantation workers	% plantation owners Using method	Perceived efficacy		
		‘Effective’	‘Semi-effective’	‘Not effective’
Firecrackers	87.0%	60.0%	30.0%	10.0%
Electric fencing	47.8%	4.5%	63.6%	31.8%
Elephant watchtowers	41.3%	26.3%	31.6%	42.1%
Non-electric fencing	34.8%	18.8%	31.3%	50.0%
Light (flashlight)	10.9%	100%	0.0%	0.0%
Noise (car, dog, voice)	6.5%	100%	0.0%	0.0%

The percentage of plantation owners that used elephant deterrence methods and their evaluation of the perceived effectiveness.

80.4% of the plantation owners were interested to try beehive fences as a new deterrence method: 32.6% mentioned that they would like help to install beehive fences now and 47.8% stated they were interested and would like to learn more.

## Discussion

Finding ways to support coexistence of humans and endangered species in areas of high human-wildlife conflict is a key challenge for conservation. In this study, we identified high levels of human-elephant conflict in the Chong Sadao district of western Thailand, with over half of the household respondents having experienced negative effects of living near elephants over the last two years, and over 95% of plantation owners reporting monthly crop raiding by elephants. Although there was general support for elephant conservation (84.6% of household respondents considered elephant conservation important), there were important differences in perceptions according to socio-economic variables and positive or negative experiences with elephants. Furthermore, there were a variety of deterrents and ideas for mitigation efforts reported by both household and plantation owners. Understanding these issues and potential solutions is a first step in mitigating human-elephant conflicts in this region of Thailand and in Asia generally.

Age of respondents and past negative experiences with elephants affected respondents’ perceptions of elephant conservation. Specifically, we found that older respondents (>35 years old) and those who had experienced a negative impact from elephants were significantly less likely to view elephant conservation as important. This may partially be due to older people having a greater likelihood of having experienced negative interactions with elephants. Indeed, we found that individuals older than 35 years had experienced more negative interactions with elephants (56.3%) as opposed to those who were younger than 35 (43.3%) (post hoc chi-square analysis: χ2 = 5.63, DF = 1, p = 0.018). In addition, individuals over 35 years of age were more likely to work in the agricultural sector (χ2 = 26.02, DF = 1, p < 0.001), an employment area directly affected by elephant crop raiding. Other factors may be at play here as well, potentially including age-based differences in education level, family responsibilities and pressures, and environmental views. Those working in agriculture, who tended to be older than 35 years of age, also were less likely to unconditionally tolerate the presence of elephants in the area. This suggests that, while it may be easier to include younger individuals in elephant conservation efforts, it also is important to engage older individuals and those who work in agriculture, as such individuals may have less supportive views overall, a longer history of interactions with elephants, and potentially more locally relevant decision-making capability.

Contrary to other studies [4,13,15], we did not find an influence of gender on expressed conservation attitudes. This may partially be due to the fact that there was not a major difference in occupation by gender. For example, roughly half of the female participants generated income through agriculture. However, men tended to make more money than women and while not statistically significant, there was not a suggestive relationship between gender and income level (post hoc chi-square analysis: χ2 = 3.536, DF = 1, p = 0.060) indicating important discrepancies that may affect conservation attitudes. A study in India suggested that women may have more negative views of elephants because they have greater responsibilities when it came to family care, household support, and prevention of elephant conflicts [15]. In Africa, it was suggested that more negative attitudes of women towards elephants were caused by their dependence on agriculture and because of the higher risk of getting in contact with elephants due to their daily duties [4]. While we did not identify any explicit gender-based differences in expressed conservation attitudes, income disparity between men and women in this area may contribute to underlying issues not identified by our study design.

Although stated support for elephant conservation did not vary by income, lower income households were less likely to support unconditional tolerance of elephants as opposed to conditional tolerance (e.g., tolerance if they could be ensured they would not destroy their crops). This makes sense, as for this group economic security is more at risk than for higher-income families. Similarly, those who work in agriculture (and thus who tended to be older than 35 years of age) were less likely to support elephant unconditional tolerance of elephants in the area. Interestingly, this result was not conflated with income level as there was not a significant relationship between income level and employment sector (post hoc chi-square analysis: χ2 = 0.397, DF = 1, p = .529). These results correspond to a study in Kenya that found people working in agriculture to be less tolerant toward elephants than pastoral people [4], a study in Congo were perceptions toward elephants of farmers were mostly negative [14] and a study in the USA about citizens’ attitudes toward wolf depredation, where occupation had the strongest influence on tolerance of these carnivores [6]. This indicates that special incentives, outreach approaches, and mitigation methods may be necessary for those at lower income levels and those working in agriculture if elephant coexistence in western Thailand is to be realized.

An important result of this study was the finding that people who perceive benefits from living near wild elephants were more likely to support their coexistence in the area, a positive association also found in India [21], Cameroon [3], Congo [14] and Kenya [4]. Perceived benefits mentioned by respondents were not just financial- they included community development, feelings of pride from hosting volunteers, and feelings of satisfaction/pride in doing conservation work. Given this result, we conducted a post hoc analysis to see if benefits of living near elephants could somewhat counteract past negative experiences with elephants. Although the subgroup sample size was small and the result was not statistically significant (post hoc chi-square analysis: χ2 = 1.082, DF = 1, p = 0.2981), individuals who had both experienced negative experiences with elephants and who also perceived benefits of living near elephants were slightly more likely to view elephant conservation as important (80.8%) as opposed to those who had only ever experienced negative effects of elephants (74.8%). Overall, our data suggests that engaging community members in conservation initiatives could substantially increase their acceptance of elephants.

Similar to studies in other areas of high human-elephant conflict [21], most plantation owners in this study reported having tried more than one deterrence method to keep elephants out of their fields, with noise and explosives being most commonly utilized and considered most effective. These methods were considered more effective than electric fences which were used by just under half of the plantation owners and generally considered to be only “semi-effective.” Well-maintained electric fences are effective in some situations [22], but their overall effectiveness depends on the maintenance [23] and design of the structure, the clearance of vegetation along the fence that can disrupt the power supply [21], and the learned behavior of the elephants to destroy or avoid the electric fence [18]. Furthermore, linear barriers such as electric fences, trenches, and other such large barriers may reduce habitat connectivity [24] and limit elephant movement with negative implications on biodiversity and ecological processes [23]. Although the sample size was relatively small, an important result of the deterrence question is that noise and lights were evaluated as very effective by the plantation owners. Traditional tools (manual torches/flashlights and noise-makers) could potentially be improved by promoting motion-activated light and noise boxes. None of the respondents mentioned the use of chili, a sustainable deterrence method that showed positive results in Kenya [25] and Zimbabwe [26].

Beehive fencing is a smaller-barrier technique that may be effective since elephants are afraid of bees [27,28]. This may be an important supplement to existing non-electric fences which, at current, were mostly considered “not effective” by plantation owners in this study (Table 4). Considering prevailing negative attitudes toward elephant conservation among plantation owners, it is interesting that 80.4% of the plantation owners expressed willingness to try beehive fences as a new deterrence method. In addition to preventing elephant damage to plantations, this method may provide villagers with the financial incentive of conservation-friendly, and therefore premium, honey production [28]. However, research is needed to analyze the effectiveness of beehive fences to deter crop-raiding elephants in Asia, and this is one area of our ongoing research.

This study has increased understanding of the challenges household members and plantation owners are facing in the Chong Sadao district of Thailand, an area of high human-elephant conflict. Ultimately, no single mitigation method can address the multi-faceted causes of the problem, which stems from increased development of original elephant habitat [29]. For deterrence solutions to have a long-term effect, they must be combined with efforts to restore natural elephant habitat, proper land use planning [25], and crop choices that are less attractive to elephants [29]. Although respondents also suggested fencing the wildlife sanctuary so that the elephants cannot get out, confining elephants in this way does not seem to be a viable long-term solution for the current elephant population considering the degradation and fragmentation of their habitat [19], carrying capacity of the area [18], reductions in genetic flow and resulting inbreeding concerns (e.g., resilience against disease), and the fact that elephants would not have access to the River Kwai Yai or corridors for migration in the event of natural disasters. Participants in this study also noted the value of forest restoration and patrol teams, adding water sources to wild elephant habitat, increased incentives for local communities, and education of local school and community groups (Table 3).

A combination of the above ideas is likely required for long-term human-elephant coexistence in the Chong Sadao district of Thailand. With continuously increasing conflicts and the limited size of Salakpra Wildlife Sanctuary, elephant conservation strategies should not only focus on restoring and maintaining available habitat but also on securing corridors to allow elephants to move to additional habitats [30,31]. Farm-based bio-fencing, e.g. beehive fences and utilizing crops that are disliked by elephants, such as ginger, lemongrass, chili, garlic and onion [32], are potential solutions that can provide additional benefits to the human population. Two encouraging outcomes of this study were that most household owners in the area feel it is important to invest in elephant conservation (84.6%) and over a third of the respondents reported perceived benefits of living near wild elephants. Overall, our results highlight the value of plantation owners receiving benefits from living near elephants. Therefore, we suggest the inclusion of plantation owners in conservation efforts—specifically, providing plantation owners with incentives and support to implement effective alternative methods to deter elephants, as the majority of the plantation owners (73.9%) are looking for such solutions. To further encourage community participation, we suggest a community-based conservation model in which stakeholders collaborate, including those working in agriculture and those at lower income levels. Although much remains to be done, these findings suggest that this area has potential to become a positive example of human-elephant coexistence.

## Supporting information

S1 Appendix Household questionnaire. The household questionnaire was conducted in October 2015 amongst 410 households on the western boundaries of Thailand’s Salakpra Wildlife Sanctuary.
(DOCX)

S2 Appendix Plantation owner questionnaire. The plantation owner questionnaire was conducted in March 2016 amongst 46 plantation owners on the western boundaries of Thailand’s Salakpra Wildlife Sanctuary.
(DOCX)

S3 Appendix Descriptive results showing socio-economic variables and past experiences with elephants (either negative experiences or perceived benefits) and residents’ attitudes toward elephant conservation and attitude toward elephant coexistence. Income was quantified as households reporting more or less than 10,000 THB (301 USD) per year.
(DOCX)

S1 Dataset Data of household and plantation owner questionnaire. (XLSX)
