# Supplementary material for: Human-elephant conflict in western Thailand: Socio-economic drivers and potential mitigation strategies
Source: PLoS One. 2018 Jun 1;13(6):e0194736. doi: 10.1371/journal.pone.0194736 (PMC5983488; doi:10.1371/journal.pone.0194736)
Supplement: S1 Appendix — The household questionnaire was conducted in October 2015 amongst 410 households on the western boundaries of Thailand’s Salakpra Wildlife Sanctuary. (DOCX) [file pone.0194736.s001.docx]

**S1: Appendix: Household questionnaire**

Questionnaire 1: October 12-19, 2015, N=410

Consent form:

“The aim of this survey is to gain and spread knowledge about wild elephants and human-elephant conflicts in Chong Sadao area in Kanchanaburi in order to launch conservation action that is self-determined and managed by the local community involved and will realize positive change. This research is being conducted as part of the graduate degree program of Antoinette van de Water under the guidance of Kevin C. Matteson at Miami University and facilitated by Siriporn Sriaram. Invitations to complete this survey have been done in person to 410 people of Chong Sadao district (Moo 1 - 7). Participation in this research is restricted to persons 18 years of age or older.

The survey will take about 20 minutes. Your participation is voluntary, you may skip exercises you do not want to participate in, and you may stop at any time. The survey does not request information that would explicitly identify you. If you inadvertently include identifying information (such as HEC accidents), such information will be removed from stored data. Only the researcher and faculty advisor will have access to individual responses. Results of the survey will only be presented publicly as aggregate summaries. If you would like to receive a report of the general results of this project, please contact Miss Siriporn Sriaram by below phone number or e-mail.

If you have any questions about this research or you feel you need more information to complete this survey, you can contact me, Antoinette van de Water, at vandewa@miamioh.edu or Miss Siriporn Sriaram, at vivi@bring-the-elephant-home.org or 088-635-4449, or my graduate advisor Kevin C. Matteson at matteskc@miamioh.edu. If you have questions or concerns about the rights of research subjects, you may contact the Research Compliance Office at Miami University at 001 513 529‐3600 or humansubjects@miamioh.edu.”

**Questions survey:**

1.              What is your gender?

❑ Male

❑ Female

❑ Other

2.      What is the name or number of your village?

3.      What is your age?

❑ 18-24 years

❑ 25-34 years

❑ 35-44 years

❑ 45-54 years

❑ 55-64 years

❑ 65+ years

4.      What is your main source of income?

❑      Agriculture

❑      Collecting forest products

❑      Government officer, medical or teacher

❑      General labor

❑      Other

5. What is your average monthly income?

❑      <10,000 THB

❑      10,000 - 20,000 THB

❑      20,000 - 30,000 THB

❑      30,000 - 50,000 THB

❑      >50,000 THB

6.      How far do you live from the border of Salakpra Wildlife Sanctuary?

❑      Inside protected area

❑      0 - 250m

❑      250m - 500m

❑      500m - 750m

❑      > 750m

7.      Over the last two years, did you or your family members experience a negative impact from human-elephant conflicts (HEC)?

❑    Yes

❑ No

             If yes, please specify:  (identifying information will be removed from stored data)

❑    Crop raiding elephants on _______________________________ (fill in crop) plantation

❑    Property damage

❑   Human injuries  (___________________________________________ please specify)

❑ Other:__________________________________________________________

8.      Did you or your family ever participate in conservation initiatives, such as homestay for volunteers or the coordination or logistics of conservation action events?

❑    Yes

❑    No

9.      Over the last two years, did you or your family members gain any benefit from living near wild elephants?

        If yes, please specify:

❑    Financial benefits through ecotourism

❑    Financial benefits through conservation jobs

❑    A feeling of pride to host volunteers

❑    A feeling of satisfaction / pride to do conservation work

❑    Community development

10.    How often did you see or hear elephants outside the protected area?

❑ Almost daily

❑ Once a week

❑ Twice a month

❑ Once a month

❑ Once in six months

❑ Once a year

        In your view, what was the reason the elephants left the protected area?

❑    Water: The river for drinking and bathing

❑    Food: Nearby plantations

❑    Encroachment

❑    Other: _________________________________________________________________

❑    Don’t know

11.    Over the last five years, do you think that HEC is stable, increasing or decreasing?

❑    Increasing

❑    Stable

❑    Decreasing

12.    Which statement describes your attitude toward elephants most accurate?

❑    I tolerate elephants in my environment

❑    I tolerate elephants in my environment if the elephants would stop destroying my

plantations

❑    I would prefer the elephants to be eradicated

13.    Do you feel it is important to invest in elephant conservation?

❑    No, because elephants are dangerous

❑    No, because elephants raid crops

❑    No, because elephants destroy property

❑    Yes, because we have a long history of living together in harmony

❑    Yes, because of their value for the ecosystem as a keystone species

❑    Yes, because elephants are the symbol of Thailand

❑    Yes, because elephants attract eco-tourists and conservation initiatives

❑ Yes, because elephants are part of our lives and culture

14.    For the following conservation initiatives, please indicate how much this is a priority to you (all answers choices can be used for all initiatives).

|  | Not a priority | Low priority | Medium priority | High priority | Essential |
| --- | --- | --- | --- | --- | --- |
| Forest restoration efforts inside the wildlife sanctuary |  |  |  |  |  |
| Forest restoration efforts at the buffer zone |  |  |  |  |  |
| Adding water sources to wild elephants habitat (check dams, reservoirs) |  |  |  |  |  |
| Fencing the wildlife sanctuary so that the elephants cannot get out |  |  |  |  |  |
| Patrol team to chase elephants back into the PA |  |  |  |  |  |
| Education of local groups and schools |  |  |  |  |  |
| Initiatives to realize benefits for local communities (ecotourism, conservation jobs) |  |  |  |  |  |

15.    Do you have any suggestions for what needs to be done to mitigate human-

elephant conflicts?

______________________________________________________________________
