# Supplementary material for: Human-elephant conflict in western Thailand: Socio-economic drivers and potential mitigation strategies
Source: PLoS One. 2018 Jun 1;13(6):e0194736. doi: 10.1371/journal.pone.0194736 (PMC5983488; doi:10.1371/journal.pone.0194736)
Supplement: S2 Appendix — The plantation owner questionnaire was conducted in March 2016 amongst 46 plantation owners on the western boundaries of Thailand’s Salakpra Wildlife Sanctuary. (DOCX) [file pone.0194736.s002.docx]

**S2: Appendix: Plantation owner questionnaire**

March 2016, N=46

1.      What crop are you growing on your plantation and what size is your plantation?

(1 rai = 1,600 m^2^)

❑      Cassava: _______________________________ rai

❑      Banana: ________________________________ rai

❑      Jackfruit: ________________________________rai

❑      Mango: ________________________________ rai

❑      Tamarind: _______________________________rai

❑      Sugarcane:______________________________ rai

❑      Other: __________________________________rai

2.      Over the last two years, how often did your plantation get raided by elephants?

❑      Almost daily

❑      Once a week

❑      Twice a month

❑      Once a month

❑      Once in six months

❑      Once a year

3.      On a yearly basis, could you estimate the amount of money you lose because of

crop-raiding?

❑      < 10,000 THB

❑      10,000–20,000 THB

❑      20,000–30,000 THB

❑      30,000–50,000 THB

❑      > 50,000 THB

4.      Besides crop-raiding, did elephants cause any other harm?

❑      Property damage

❑      Human injuries  (_____________________________________   please specify)

❑      Other:_______________________________________________________

5.      What method(s) to deter the elephants have you tried and how effective was this?

❑      Electric fencing:                                effective/semi-effective/not effective

❑      ______________ fencing:                effective/semi-effective/not effective

❑      Elephant watch towers:                   effective/semi-effective/not effective

❑      Firecrackers:                         effective/semi-effective/not effective

❑      Other:_________________:    effective/semi-effective/not effective

6.      Which statement describes your attitude toward elephants most accurately?

❑      I tolerate elephants in my environment, even if they destroy my plantation

❑      I would tolerate elephants in my environment if they would stop destroying my

plantations

❑      I would prefer elephants to be eradicated

❑      Other:_______________________________________________________

7.      Which statement describes your vision of the future most accurately?

❑      I will continue with my plantation the way I am working now

❑      If the crop-raiding doesn’t stop, I’ll need to sell my plantation

❑      I need to find a better method to stop elephants raiding my plantation

❑      I would consider changing to a crop elephants don’t like

❑      Other:_______________________________________________________

8.      On the attached leaflet you can read about a new strategy to prevent crop-raiding:

beehive fencing. What are your initial thoughts about using beehives to deter elephants?

❑      Please help me to install a beehive fence at my plantation now

❑      I am interested and would like to learn more

❑      I don’t believe this will work

❑      I have no interest to try a new method

9.      If you are interested in beehive fences, then what kind of support do you need?

❑      Training in beekeeping

❑      Training in the construction of beehive fences

❑      Financial support for the initial investment

❑      A market for the honey

❑      Other:_______________________________________________________

10.    To monitor human–elephant conflicts you can soon download the new app

HECTOR on smartphones. To prepare for the launch of HECTOR, could

you please let us know what type of phone you have?

❑      Smartphone, type: _____________________________

❑      Regular mobile phone, type: ______________________

❑      I don’t use a mobile phone

11.    Could you also let us know how often you have access to 3G, 4G, or a WIFI

network?

❑      Almost always

❑      A few hours a day

❑      At least once a day

❑      A few times a week

❑      Hardly ever

12.    Are you willing to join the citizen science team for new technologies to reduce crop

raiding?

❑      Yes, I am interested in using a new technology to reduce crop-raiding

❑      I am interested to become a HEC inspector, but I have to gain more knowledge

about the technology

❑      I know someone who would be willing to become a HEC inspector

❑      I have no interest to try a new technology
