# Supplementary material for: Human-elephant conflict in western Thailand: Socio-economic drivers and potential mitigation strategies
Source: PLoS One. 2018 Jun 1;13(6):e0194736. doi: 10.1371/journal.pone.0194736 (PMC5983488; doi:10.1371/journal.pone.0194736)
Supplement: S3 Appendix — Income was quantified as households reporting more or less than 10,000 THB (301 USD) per year. (DOCX) [file pone.0194736.s003.docx]

**S3 Appendix. Descriptive results showing socio-economic variables and past experiences with elephants (either negative experiences or perceived benefits) and residents’ attitudes toward elephant conservation and attitude toward elephant coexistence.** The questionnaire was conducted in October 2015 amongst 410 households on the western boundaries of Thailand’s Salakpra Wildlife Sanctuary, home of an estimated 181 Asian elephants. Income was quantified as households reporting more or less than 10,000 THB (301 USD) per year.

| Summary of responses by variables | | Attitude toward elephant coexistence | | Attitude toward elephant coexistence | | |
| --- | --- | --- | --- | --- | --- | --- |
|  |  | Not important | Important | Eradicate | Conditional tolerate | Tolerate |
| Gender | Female | 15.6% | 84.4% | 33.3% | 38.6% | 28.0% |
|  | Male | 15.2% | 84.8% | 35.5% | 37.6% | 26.9% |
| Employment sector | Agricultural | 17.2% | 82.8% | 50.0% | 42.6% | 7.4% |
|  | Non-agricultural | 14.6% | 85.4% | 29.4% | 36.8% | 33.9% |
| Age | <35 yrs | 8.3% | 91.7% | 25.6% | 38.0% | 36.4% |
|  | 35 and older | 18.4% | 81.6% | 38.0% | 38.0% | 23.9% |
| Income | <10k THB | 15.1% | 84.9% | 34.7% | 42.1% | 23.1% |
|  | 10k+ THB | 16.0% | 84.0% | 34.0% | 31.5% | 34.6% |
| Experienced a negative impact from elephants? | Yes | 22.0% | 78.0% | 28.5% | 47.2% | 24.3% |
|  | No | 8.3% | 91.7% | 40.4% | 28.2% | 31.4% |
| Received benefits from elephants? | Yes | 16.00% | 84.00% | 17.6% | 43.7% | 38.7% |
|  | No | 15.30% | 84.70% | 44.3% | 34.0% | 21.7% |
